# Neurocognitive dysfunction and pharmacological intervention using guanfacine in a rhesus macaque model of self-injurious behavior

**DOI:** 10.1038/tp.2015.61

**Published:** 2015-05-19

**Authors:** Z T Freeman, K A Rice, P L Soto, K A M Pate, M R Weed, N A Ator, I G DeLeon, D F Wong, Y Zhou, J L Mankowski, M C Zink, R J Adams, E K Hutchinson

**Affiliations:** 1 Department of Molecular and Comparative Pathobiology, Johns Hopkins University School of Medicine, Baltimore, MD, USA; 2 Division of Veterinary Resources, National Institutes of Health, Bethesda, MD, USA; 3 Department of Psychiatry and Behavioral Sciences, Johns Hopkins University School of Medicine, Baltimore, MD, USA; 4 Department of Behavioral Psychology, Kennedy Krieger Institute, Baltimore, MD, USA; 5 Department of Radiology and Radiological Science, Johns Hopkins School of Medicine, Baltimore, MD, USA; 6 Department of Neuroscience, Johns Hopkins School of Medicine, Baltimore, MD, USA

## Abstract

Self-injurious behavior (SIB) is a common comorbidity of psychiatric disorders but there is a dearth of information about neurological mechanisms underlying the behavior, and few animal models exist. SIB in humans is characterized by any intentional self-directed behavior that leads to wounds, whereas in macaques it is not always accompanied by wounds. We describe a cohort of rhesus macaques displaying SIB as adults, in which changes within the central nervous system were associated with the SIB. In these macaques, increases in central nervous system striatal dopamine (DA) receptor binding (BP_ND_) measured by positron emission tomography (PET) [11C]raclopride imaging correlated with severity of wounding (*r*_s_=0.662, *P*=0.014). Furthermore, utilizing standardized cognitive function tests, we showed that impulsivity (stop signal reaction time, SSRT) and deficits in attentional set shifting (intra-/extradimensional shift) were correlated with increased severity of SIB (*r*_s_=0.563, *P*=0.045 and *r*_s_=0.692, *P*=0.009, respectively). We also tested the efficacy of guanfacine, an α_2_A adrenergic agonist that acts to improve postsynaptic transmission of neuronal impulses, in reducing SIB. A subset of these animals were enrolled in a randomized experimenter-blinded study that demonstrated guanfacine decreased the severity of wounding in treated animals compared with vehicle-only-treated controls (*P*=0.043), with residual beneficial effects seen for several weeks after cessation of therapy. Animals with the highest severity of SIB that received guanfacine also showed the most significant improvement (*r*_s_=−0.761, *P*=0.009). The elevated PET BP_ND_ was likely due to low intrasynaptic DA, which in turn may have been improved by guanfacine. With underlying physiology potentially representative of the human condition and the ability to affect outcome measures of disease using pharmacotherapy, this model represents a unique opportunity to further our understanding of the biology and treatment of SIB in both animals and humans.

## Introduction

In humans, self-injurious behavior (SIB) is a high-impact manifestation of underlying psychiatric disorders, with outcomes ranging from social ostracism to severe physical injuries and/or death.^1^ SIB in humans is characterized by any intentional self-directed act that leads to physical harm.^2, 3^ The development of novel targeted therapeutics is impeded by a dearth of understanding of the physiological mechanisms underlying SIB and lack of appropriate models for testing of interventions.^4^ Although the biological connections between psychological abnormalities, their precipitating events and their manifestation as SIB are not well defined, evidence from human disease and animal models suggests neurochemical changes contribute significantly. Alterations in the brain dopaminergic system, with its role in reward-driven learning^5^ and working memory,^6^ is one proposed mechanism by which early experience and trauma could manifest as later psychiatric diseases and subsequent SIB.

In humans, the most compelling case of alterations in dopamine (DA) contributing to SIB is in Lesch–Nyhan syndrome, a genetic disorder characterized by abnormal purine metabolism resulting in impaired cognitive development and function, as well as distinctive patterns of SIB.^7, 8^ In addition to difficulty with certain cognitive tasks, especially those associated with working memory, Lesch–Nyhan syndrome patients have been demonstrated to have decreased dopaminergic function,^9^ DA transporter,^10^ and dopaminergic nerve terminals and cell bodies.^11^ In rodent models, targeted disruption of the dopaminergic system by administration of methamphetamine or 6-hydroxy-DA has been used to model human SIB.^12, 13^

The connection between altered cognitive function and SIB has long been established. The strong association between SIB and mental retardation has been documented by work beginning in the late 1970s.^14^ Among adult patients with a diagnosis of schizophrenia or borderline personality disorder, severity of SIB is positively correlated with errors on multiple cognitive function assessments.^15^ In addition to correlating with greater general cognitive impairment and decreased speech ability among children with autism, elevated severity of SIB is also associated with increased levels of impulsivity.^16^ Similarly, rhesus macaques with SIB have been demonstrated to exhibit cognitive alterations, including longer time to extinction of a learned behavior than controls.^17^

Animal models hold the potential to further elucidate the pathobiology underlying SIB and its comorbid disorders and to serve as a means for testing novel therapies. Whereas rodent models of SIB have elucidated potentially related neurochemical changes, their deviation from the human condition in relevant behavioral manifestations, anatomy and physiology may limit their usefulness.^18^ Nonhuman primates (NHPs) exhibiting SIB are more suitable because of their phylogenetic proximity to humans and resultant analogy in relevant behavioral repertoire, anatomy and physiology. SIB occurs sporadically in NHP housed in research settings, with reported prevalence ranging from 5 to 25% among individually housed rhesus macaques.^19, 20, 21^ SIB animals are usually identified by direct observation of the behavior or incidences of self-injurious wounds documented in the veterinary record.^20^ SIB in NHPs is defined by self-directed behavior including hair plucking, head banging and self-biting, each of which may or may not lead to tissue damage.^19^ Single housing at an early age, duration of single housing, number of blood draws/sedations, sex and nursery rearing have been document as risk factors associated with the development of SIB in NHPs.^20^ Whereas many characteristics of NHP SIB make them a potentially useful animal model, the sporadic nature and late onset of detectable behavior, which is frequently seen only after animals have already been assigned to unrelated studies, have historically made the formation of a suitable study cohort difficult. Nonetheless, reports of treatment for NHP SIB largely mirror the human literature, both in the specific treatments used and their mixed or inconsistent results.^22, 23^

Adrenergic α2 agonists are one class of drug that has shown promise in both the human and NHP literature. In one case report, the α2 agonist clonidine was demonstrated to alleviate SIB with wounding in a young girl.^24^ Guanfacine, a preferential α2A receptor agonist originally used to treat hypertension, and more recently to treat ADHD, was reported in another case study to decrease wounding in two rhesus macaques and one baboon with SIB.^25^

Herein we describe a cohort of 19 rhesus macaque males, 7 years of age (YoA) that developed a high incidence of self-inflicted wounds. We hypothesized that the severity of SIB in these rhesus macaques would relate to measurable neurocognitive dysfunction similar to that described in humans with SIB, and that this dysfunction would develop before and relate to the clinical severity of wounds. We further hypothesized that pharmacological intervention using guanfacine, an α2A adrenergic agonist, would reduce both severity and frequency of wounding.

## Materials and methods

### Animals

Nineteen adult male rhesus macaques (*Macaca mulatta*), 7 YoA and weighing an average of 10.7 kg (range of 8.3–14.9 kg), were housed in compliance with the Animal Welfare Act Regulations and the Public Health Service Policy on the Humane Care and Use of Laboratory Animals at Johns Hopkins University facilities that are accredited by the Association for Assessment and Accreditation of Laboratory Animal Care International. The animals were singly housed for the duration of the study to allow for administration of therapy and to ensure that any wounds present were self-inflicted. The animals were maintained on a 14-h light and 10-h dark schedule, fed a commercial macaque diet (Harlan, Indianapolis, IN, USA), given water *ad libitum* and provided with environmental enrichment (for example, fruit/vegetables) daily.

Our chosen dependent measure for the purpose of this study was wound severity and distribution in the form of wound score (WS). Although we have demonstrated that for animals with wounds, this WS is related to biting behavior,^26^ our own data and others^19^ have suggested that some animals may bite themselves quite frequently without causing wounds. Thus, animals without wounds may have quite severe behavioral pathology, or none at all, and our chosen measure would not be able to distinguish between the two. For this reason, only animals with non-zero WSs during baseline conditions and complete historical data sets for stop signal reaction time (SSRT), intra-/extradimensional (ID/ED) and [11C]raclopride binding positron emission tomography (PET) were included in the retrospective analysis. For this same reason, coupled with the fact that previous studies have found some SIB ‘treatments' to actually exacerbate wounding,^22^ we wanted to include a small number of animals with no baseline wounds in our treatment arm of the study, thus we could ensure that guanfacine does not have a significant negative effect on such animals, such as might result from disinhibition. Of the 19 original animals in our cohort, six showed no baseline wounds despite documented histories of self-biting behavior. We randomly selected three of these six animals for inclusion in the guanfacine treatment arm of this study, and the other three animals were transferred to another study before beginning the treatment.

The animals had served in a study examining the effects of early-life administration of stimulant drugs and had been exposed to one of three treatment regimens (that is, no drug, dl-amphetamine or methylphenidate) between the ages of 2 and 4.5 years;^27^ they then had been provided access to oral cocaine and/or alcohol from ages 5.5 to 6.5 years (unpublished data). This experiment required that the animals be singly housed in cages, yet still allowing for visual and auditory contact, which began when the animals were between 458 and 660 days of age. The first clinical record of wounds secondary to SIB occurred when the animals were 6 YoA, with the prevalence of wounds and/or observed self-biting reaching 100% by 7 YoA. Importantly, there were no significant differences in wounding incidence or severity as a function of previous experimental treatment group (*n*=19, Kruskal–Wallis *P*=0.107).

### Wound scores

Objective measurement of SIB can be quite difficult and labor intensive. Although 24-h direct observation is the ideal way to measure the behavior, in the majority of both human and NHP cases this is not possible owing to practical limitations of having observers present at all times and/or the high manpower costs of reviewing remotely obtained video. Previous reports examining SIB in monkeys have thus relied upon representative sampling for observations, but typically report relatively small samples (for example, six total hours in 4 weeks of a treatment condition).^23, 28^ In cases where wounding is present, however, it is possible to measure the outcomes of SIB in terms of tissue trauma. Iwata *et al.*^29^ and Grace *et al.*^30^ have described using WSs and locations as a means of quantifying SIB in human cases where direct observation is difficult. We adapted this method to NHPs to obtain WSs, with consideration to clinically relevant differences in wound severity and distribution. We have previously shown that our WS system is associated with observed SIB behavior, and represents the severity and distribution of wounds in individual macaques.^26^

To obtain WSs, animals were sedated with 10–20 mg kg^−1^ ketamine HCl intramuscularly, and physical examinations were performed at 2-week intervals. Wounds were identified individually, photographed and mapped on the body of each animal. Individual wounds were scored based on severity according to the following criteria:

1. A pinpoint or pinprick wound, not penetrating dermis, round in shape and no bigger than 5 mm in diameter
2. Any wound not penetrating dermis and >5 mm in length or diameter
3. A wound penetrating dermis, round in shape, no bigger than 5 mm in diameter
4. A wound penetrating dermis and >5 mm in length or diameter

Each quadrant (one quarter of the body, separated by midline sagitally and at the level of the umbilicus transversely) was assigned a score corresponding to the most severe wound found in that quadrant. All quadrant scores were summed to give a total WS (minimum 0 and maximum 16). Wound scoring was conducted by observers blinded to experimental group assignments. A veterinarian, who was blinded to the conditions of the trial, was responsible for all clinical decisions pertaining to wounds present on individuals. No wounds observed during this study were deemed to require veterinary intervention

### Stop signal reaction time

The SSRT procedure is considered to measure the ability to inhibit an action (that is, inhibitory control). SSRT was tested from 1000 hours to 1100 hours 3 days per week as previously described.^27^ Animals were trained on a pellet delivery apparatus with a computer touch screen and a lighted lever. The monkeys learned to hold the lever, then release and touch a white key, but not a red key, when it appeared on the screen. Probe trials then measured how quickly an animal could adjust its response if the white key turned to red in the time between releasing the lever and touching the screen.

### ID/ED set shifting task

The ID/ED task assesses attentional set shifting^31^ and was performed as previously described.^27^ The ID/ED task was conducted at the same time of day for a given animal (1100 hours or 1400 hours) and typically took 4.7 days, on average, to complete. All other behavioral testing was suspended during ID/ED testing. The number of errors during each task and the average time to completion of a trial (latency time) were calculated. This task was performed using a touch-screen computer and consisted of up to eight discrimination-learning stages wherein one of two stimuli, consisting of a background solid shape and foreground line, on the screen is reinforced.^31, 32^ The ID shift occurs when the shape/line pairs change but the same aspect that was previously rewarded (for example, shape) is still reinforced. The ED shift occurs when the shape/line pairs change again, but the reinforced stimulus is shifted from the background shape to one of the foreground lines. Errors in either stage were summed by counting any incidence of touching the shape not associated with reinforcement. ID/ED errors represent the number of errors, ID and ED stages to reach a preset success criterion (12 correct responses in 15 trials).

### PET magnetic resonance imaging

Parametric images of binding potentials were generated from [11C]raclopride dynamic PET to determine DA D2/D3 receptor binding. Magnetic resonance imaging for regions of interest, including dorsal striatum, caudate nucleus and putamen was performed as previously documented 1 month before the [11C]raclopride scan.^27^ Briefly, animals were anesthetized and underwent PET scans on a high-resolution research tomography PET scanner. At the beginning of the scan, [11C]raclopride was administered as a bolus and subsequently maintained as continuous infusion. D2/D3 receptor binding potential (BP_ND_) was calculated using a simplified tissue reference model^33^ for each region of interest identified in the earlier magnetic resonance imaging scans.

### Guanfacine treatment

Animals were stratified into four groups according to baseline WSs, and one animal was randomly selected from each severity group to serve as a vehicle-only-treated control. After separation into guanfacine (*n*=12) and vehicle-only (*n*=4)-treated groups, animals initially received oral food vehicle twice daily for 4 weeks. The animals then received twice-daily oral administration (~0800 hours and 1700 hours) of either 5 mg of guanfacine (Watson Pharmaceuticals, Parsippany, NJ, USA) delivered in the vehicle (Skittles, Wrigley, McLean, VA, USA) or vehicle alone for 4 weeks. Animals were observed an additional 4 weeks after the cessation of therapy with continued twice-daily administration of vehicle. Three animals in the guanfacine-treated group were removed from the study as a result of refusal to accept oral treatment, and their results were discarded. After the end of the initial study design, WSs were collected at 4–6 week intervals until wounding returned to baseline levels.

### Data analysis

All statistical analyses were performed using GraphPad Prism 6.0 (GraphPad Software, San Diego, CA, USA) or Stata (StataCorp, College Station, TX, USA), and *P*-values <0.05 were considered significant. Correlations were analyzed using a Spearman's rank test. The WSs of animals treated with guanfacine or vehicle only were compared across three treatment phases (pretreatment, treatment and posttreatment) using mixed model linear regression with repeated measures. Change in WSs from before and after treatment for guanfacine versus vehicle-only-treated controls were compared using a Mann–Whitney test.

## Results

### Stop signal reaction time

**Figure 1:**
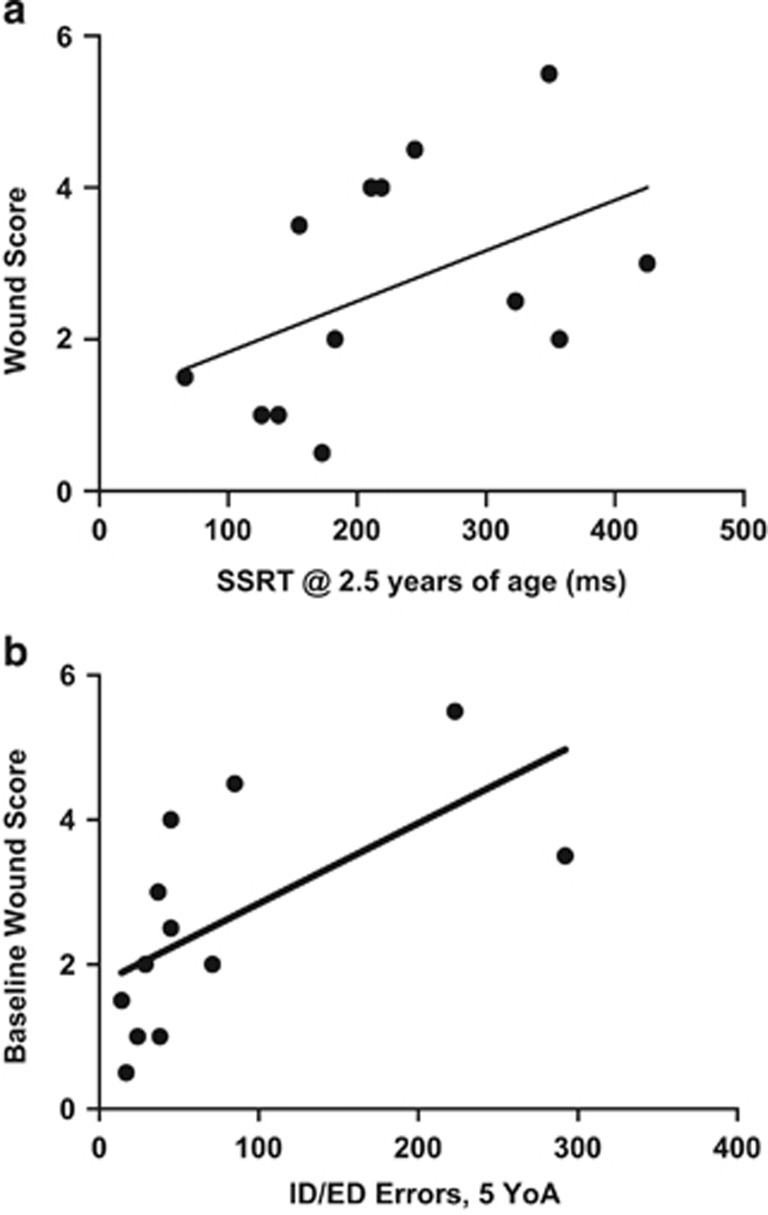
Decreased performance on cognitive tasks as a juvenile correlates with increased severity of self-injurious behavior (SIB) in adulthood. (**a**) Baseline wound score (WS) at 7 YoA was positively correlated with impulsiveness as measured by stop signal reaction time (SSRT) at 2.5 YoA. (*r*_s_=0.563, *P*=0.045). (**b**) Baseline WS at 7 YoA was positively correlated with deficits in attentional set shifting as measured by intra-/extradimensional (ID/ED) errors at 4.5 YoA (*r*_s_=0.789, *P*=0.002).

Impulsivity, previously associated with SIB,^16^ has been measured using the SSRT procedure in both humans^34^ and laboratory animals such as rodents and monkeys.^35^ Each individual's average WS at 7 YoA was compared with SSRT recorded at 2.5 YoA and found to be positively correlated (*n*=13, *r*_s_=0.563, *P*=0.045; Figure 1a), thereby implying a relationship between impulsivity early in life and later SIB severity.

### ID/ED performance

The ID/ED task assesses attentional set shifting and has been used to reflect executive function and general cognitive performance.^31, 36^ We analyzed the total number of errors to reach success criterion (12 correct responses out of the most recent 15 trials) on the ID and ED shift tasks performed on a touch-screen apparatus by each animal at 2.5, 3, 4 and 4.5 YoA. ID/ED performance did not vary significantly among previous treatments (amphetamine treated, methylphenidate treated and untreated control) at any time point (*P*=0.6293). Comparing subjects' ID/ED errors at all time points to their WS at 7 YoA revealed a significant positive correlation (*n*=13, *r*_s_=0.670, *P*=0.014). Based upon this, we compared baseline WS and ID/ED errors at successive ages separately (2.5, 3, 4 and 4.5 YoA). The number of ID/ED errors at ages 2.5 and 3 had no discernable relationship with wounding (*r*_s_=0.243, 0.155 and *P*=0.421, 0.612, respectively). However, at 4 YoA, the correlation between number of ID/ED errors committed and later WS showed a trend toward a positive correlation (*r*_s_=0.480, *P*=0.099), with this relationship becoming statistically significant at 4.5 YoA (*r*_s_=0.789, *P*=0.002; Figure 1b). These results imply a relationship between cognitive deficits, developing during the juvenile period, and the severity of SIB in adulthood.

### DA D2/D3 receptor binding potentials compared with SIB severity

**Figure 2:**
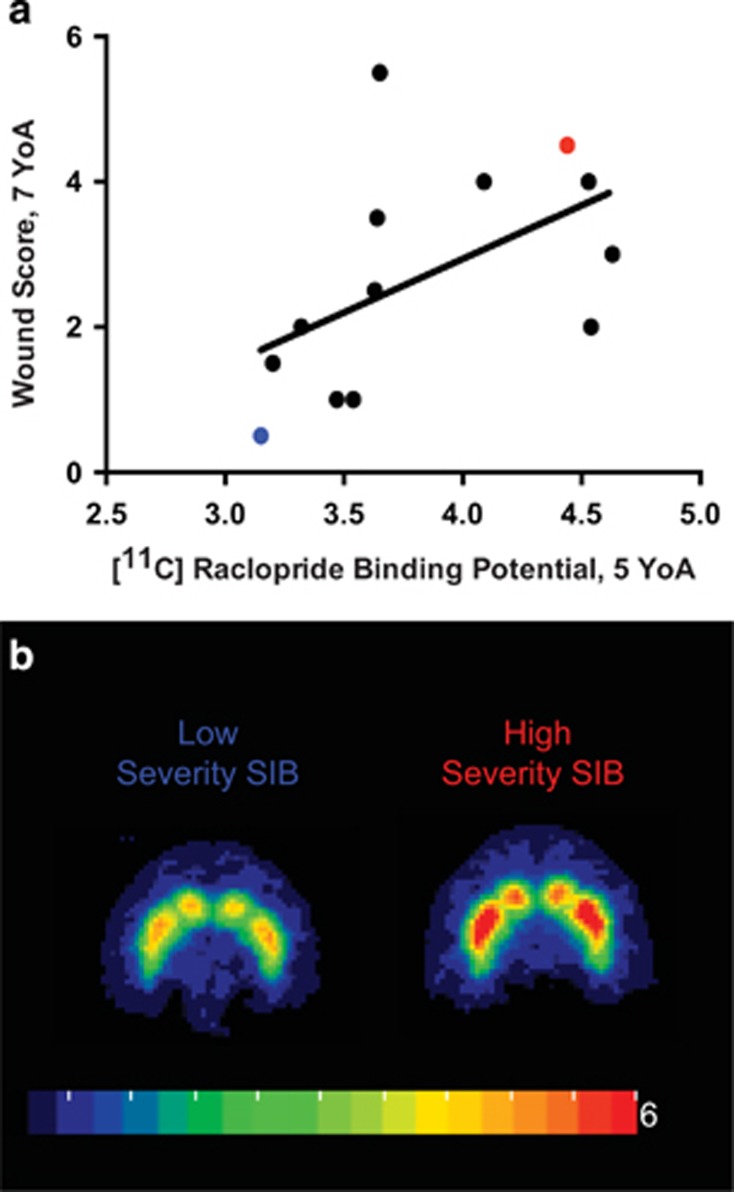
Dopamine receptor levels correlate with severity of self-injurious behavior (SIB) later in life. (**a**) [11C]raclopride positron emission tomography (PET): Dopamine D2/D3 receptor binding potentials (D2/D3 BP_ND_) at 5 YoA were positively correlated with average baseline wound score performed at 7 years of age for each of the three regions (dorsal striatum nucleus *r*_s_=0.662, *P*=0.014; putamen nucleus Spearman *r*=0.615, *P*=0.025 and caudate nucleus *r*_s_=0.566, *P*=0.044). (**b**) A representative image of dorsal striatal [11C]raclopride PET D2/D3 BP_ND_ for low- and high-severity wounding individuals (red regions indicate higher binding potential). Blue and red dots represent corresponding low- and high-severity SIB from Figure 2a.

DA has been previously implicated as a potential mechanism for some human disorders resulting in SIB;^7, 9^ therefore, DA D2/D3 receptor binding potentials (BP_ND_) for three regions of interest known to have high densities of dopaminergic synapses - the dorsal striatum, caudate nucleus, and putamen - were calculated based on [^11^C]raclopride PET scans of animals performed at 5 YoA. We compared D2/D3 receptor BP_ND_ with each individual's average WS representing severity of SIB at 7 YoA (*n*=13) and found a positive correlation for each of the regions of interest (dorsal striatum: *r*_*s*_=0.662 *P*=0.014, caudate nucleus: *r*_*s*_=0.566 *P*=0.044, putamen: *r*_*s*_=0.615 *P*=0.025, Figure 2a). Figure 2b shows representative dorsal striatal [^11^C]raclopride PET–magnetic resonance imaging images of an individual with low WS and low D2/D3 BP_ND_ (blue), and an individual with high WS and high D2/D3 BP_ND_ (red, individual values highlighted in Figure 2a). These data suggest a relationship between DA D2/D3 binding potential during subadulthood and SIB severity later in life.

### Effect of guanfacine on SIB

**Figure 3:**
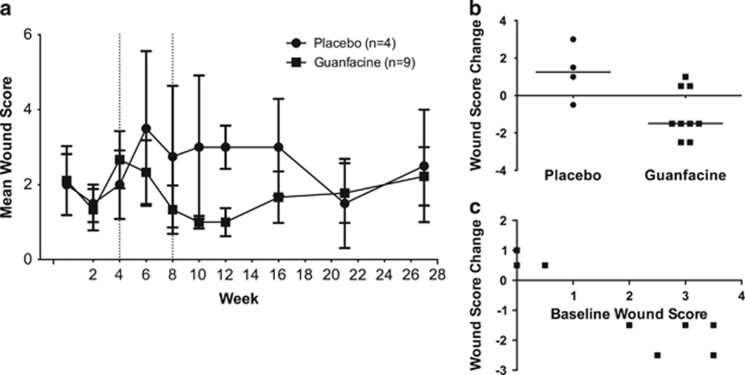
Guanfacine therapy decreases self-injurious behavior (SIB). (**a**) Wound scores (WSs) recorded throughout the experiment are showed. Treatment condition was a significant factor in determining WS over the course of the experiment (the beginning and end of the treatment phase are demarcated by dashed lines at weeks 4 and 8, *P*=0.035). (**b**) Guanfacine-treated animals experienced a significant decrease in WS from pre- to posttreatment periods when compared with vehicle-only-treated animals (Mann Whitney, *P*=0.021). (**c**) In animals treated with guanfacine, baseline WS was inversely correlated with the change in WS after treatment, that is, animals that were worse before treatment had larger response to treatment (*r*_s_=−0.7606, *P*=0.009).

To evaluate the effect of guanfacine, self-injurious macaques were treated with 5 mg guanfacine (*n*=9) or vehicle only (*n*=4) by mouth twice daily after 4 weeks of baseline measurements and continuing through week 8. WSs were recorded at 2-week intervals from week 0 through week 12, then at 4–6 week intervals, thereafter until the effects of guanfacine treatment were observed to have waned (Figure 3a). A divergence of WSs in guanfacine-treated animals versus controls emerged during the 4 weeks of treatment (study weeks 6 and 8), with WSs decreasing from baseline in the guanfacine group and increasing in the control group. Using a repeated measures mixed model linear regression, we found that treatment condition was a significant factor contributing to WS over the course of the experiment, with guanfacine-treated animals having less clinically significant wounding than their vehicle-only-treated counterparts (*P*=0.035; Figure 3a). In study weeks 10 and 12, the month after the cessation of treatment, guanfacine-treated animals had significantly lower wounds scores than during vehicle-only treatment (*P*=0.049; Figure 3a), as well as a significant improvement when compared with vehicle-only-treated controls (*P*=0.021; Figure 3b). To quantify the effect of treatment, each individual's average WS in the two time points before treatment was subtracted from that animal's average WS in the two time points following the cessation of treatment. Guanfacine-treated animals had a significantly greater decreases in post- versus pretreatment WSs when compared with vehicle-only-treated animals. This effect of guanfacine persisted until week 21, 13 weeks after cessation of therapy. WSs for each treatment group reconverged at this point and remained equivalent 6 weeks later (Figure 3a). Animals with more severe wounding had greater improvements with treatment, that is, the change in WS from baseline to treatment was also negatively correlated with pretreatment WS (*r*_s_=−0.76, *P*=0.009; Figure 3c).

## Discussion

In our cohort of rhesus macaques, poorer performance on cognitive tasks early in life was associated with increased severity of SIB in adulthood. Impulsivity in these animals, as measured by SSRT at 2.5 YoA, was positively correlated with WS at 7 YoA. This finding mirrors the human condition; impulsive behavior in children is associated with SIB in Cornelia de Lange, Fragile X, Lowe and Prader–Willi syndromes,^37^ as well as autism spectrum disorders.^16^ Likewise, borderline personality disorder patients with SIB demonstrate high levels of impulsivity compared with normal controls.^38^ Impaired performance during attentional set shifting (ID/ED) tasks was also correlated with increased severity of SIB in our animals. Similarly, degree of cognitive impairment correlates with SIB in human pediatric cases^37, 39^ and in adult schizophrenia and borderline personality disorder.^15^ Interestingly, the relationship between this measure of cognitive performance and SIB appeared to develop as our animals matured, trending toward correlation at 4 YoA and becoming significant at 4.5 YoA. Similarly, alterations in impulsivity have been demonstrated to be a significant predictor of SIB in children with autism spectrum disorder.^39^

We also observed that DA D2/D3 receptor binding by [^11^C]raclopride at 5 YoA positively correlated with wounding severity at 7 YoA, suggesting that increased D2/D3 receptor availability is correlated with increased wound severity. Increased receptor binding potential could reflect decreases in endogenous ligand. In Parkinson's disease, which is characterized by a decrease in central nervous system DA, one study demonstrated increased D2/D3 receptor binding by [^11^C]raclopride in affected patients compared with age-matched controls.^40^ A similar increase in D2/D3 receptor binding and decreased DA has been observed *ex vivo* in the brains of Lesch–Nyhan syndrome patients.^41^ Our findings of increased D2/D3 receptor binding therefore suggest that these macaques may have had decreases in intrasynaptic DA or increases in receptor expression at 5 YoA, which were not present at other time points but nevertheless predicted the severity of the SIB at 7 YoA. This mirrors our attentional set shifting results, which also developed a relationship with SIB only as the monkeys aged. Indeed, although [^11^C]raclopride binding was not directly correlated with ID/ED errors in our animals, decreases in DA function have been correlated with impaired performance on attentional set shifting tasks in humans.^42^ In addition, research into Parkinson's disease^43^ and HIV-associated cognitive decline^44^ suggests that damage to the dopaminergic system may be permanent, which could explain the relationship between temporally distant measures in our monkeys. We currently have only historic cognitive and D2/D3 receptor binding potential data to compare to more contemporary WSs in these animals, but determination of whether changes in these systems and their relationship to SIB severity are permanent represents an important direction for future studies.

Guanfacine significantly decreased the wounding associated with SIB in our monkeys, having the greatest effect in those animals with the highest WSs. Although previous case reports have suggested the potential for guanfacine to treat SIB in both children and NHPs, this is the first report of a controlled prospective study demonstrating its efficacy in alleviating SIB. Guanfacine, a preferential α2A agonist, improves neurotransmission by inhibiting postsynaptic signal disruption mediated by cyclic adenosine monophosphate.^45^ It has previously been demonstrated that guanfacine improves impulsivity in rats in a similar manner as dopaminergic agonists,^46^ and improves cognitive performance on working memory tasks via enhanced function of the prefrontal cortex.^47^ In addition, guanfacine is known to alter DA signaling^48^ and decrease the symptoms of Tourette's disorder in children, a disease characterized by decreased DA function in the striatum.^49^ It thus seems possible that guanfacine decreased SIB severity via temporary correction of the changes we found in the dopaminergic system. We also found that guanfacine had a lasting effect past the cessation of drug administration. The elimination half-life of guanfacine is 13–14 h in children and 17 h in adults, suggesting that its pharmacological effects should not last beyond 2–3 days after cessation of drug administration.^50^ Despite this, guanfacine-treated animals were not observed to return to similar levels of wounding as vehicle-only-treated controls until 13 weeks after the end of treatment (study week 21). One potential explanation for this latent effect in the face of a short drug half-life is the potential that improvement was not caused by the drug itself, but by a withdrawal effect. However, this would not explain why treatment animals were already trending toward improvement in comparison with vehicle-only-treated controls during treatment weeks 6 and 8. A more likely explanation would be that the correction of DA function led to more persistent downstream effects. With DA's known role in reward-based learning, it could be that there was a temporary correction of whatever aberrant learning process leads the animals to experience SIB as a rewarding event. This ‘relearning' while on treatment could then theoretically outlast the effects of the drug itself. Treatment with extended release naltrexone was shown to have similar positive effects and outlast the duration of the drug treatment in another study of rhesus macaques with SIB.^23^ Naltrexone's efficacy is presumably owing to disruption of the reinforcing effects of SIB by blockade of the endogenous opioid release accompanying injury, and a similar ‘relearning' process could help explain the effects seen after treatment. This highlights one potential area for future study: the examination of DA and cognitive function both during and after treatment with guanfacine.

NHPs have not been extensively utilized to model human SIB for a variety of practical and ethical reasons. The prospect of purposefully inducing SIB in NHPs for modeling purposes would be ethically questionable, and assembling a large enough cohort of animals with spontaneously occurring disease is made difficult by the sporadic nature of SIB in macaques, and the fact that it is often not detected until after the age at which animals are typically assigned to other experimental protocols. However, our findings support the notion that the pathophysiology of SIB is similar in humans and NHPs, and an existing cohort such as the one we worked within for this study represents a unique opportunity to explore underlying mechanisms and potential treatments of SIB. Although there was no significant relationship between historical drug therapies from the animals' original study or administration of drugs on behavioral or neuroimaging data,^27^ we cannot completely rule out what effect previous psychostimulant drug administration may have had on the development of SIB within this cohort. Further work is needed to compare these SIB macaques with humans and other cohorts of SIB and normal macaques to determine how the pathophysiology compares among these groups. SIB in humans with intellectual deficits has been treated with some success by increasing functional serotonin levels, whether by treatment with selective serotonin reuptake inhibitors^51^ or by dietary supplementation with serotonin containing foods.^52^ Similarly, SIB in rhesus macaques has been successfully treated via hyper-supplementation of tryptophan, serotonin's amino-acid precursor^28^ or by selective serotonin reuptake inhibitors.^53^ Naltrexone has been a drug of much interest in treating human SIB following Sandman's work linking beta-endorphin cleavage and SIB, although the results in subsequent clinical trials have been mixed.^51, 54^ Alterations in beta-endorphin metabolism have also been suggested in NHPs with SIB,^55^ and the recent report of successful treatment of macaque SIB with long-acting naltrexone further supports this hypothesis.^23^

The present study adds to and expands the growing body of literature, suggesting that SIB in humans and NHPs may arise from similar mechanisms and thus share potential therapeutic targets. Herein, we have demonstrated that NHPs exhibiting SIB as adults have decreased DA function and display cognitive deficits as juveniles, both of these have also been seen in the context of human SIB.^9, 10, 11, 15^ We have also shown that treatment with guanfacine, an α2A agonist similar to one previously utilized to treat human SIB, was also effective in treating SIB in our cohort of macaques.^24^ The increased BP_ND_ found in SIB is likely related to the decreased intrasynaptic DA, which in turn may have been ameliorated by treatment with guanfacine. These results hold the promise to help elucidate the problem of SIB not only in captive NHPs but potentially also in their human counterparts as well.
